# Diversity, Functions and Antibiotic Resistance of Sediment Microbial Communities From Lake Geneva Are Driven by the Spatial Distribution of Anthropogenic Contamination

**DOI:** 10.3389/fmicb.2021.738629

**Published:** 2021-10-18

**Authors:** Emilie Lyautey, Chloé Bonnineau, Patrick Billard, Jean-Luc Loizeau, Emmanuel Naffrechoux, Ahmed Tlili, Edward Topp, Benoît J.D. Ferrari, Stéphane Pesce

**Affiliations:** ^1^INRAE UR RiverLy, Villeurbanne, France; ^2^INRAE, Université Savoie Mont Blanc, CARRTEL, Thonon-les-Bains, France; ^3^Université de Lorraine, CNRS, LIEC, Nancy, France; ^4^Department F.A. Forel for Environmental and Aquatic Sciences, University of Geneva, Geneva, Switzerland; ^5^EDYTEM, CNRS, Université Savoie Mont Blanc, Chambéry, France; ^6^Eawag, Swiss Federal Institute of Aquatic Science and Technology, Dübendorf, Switzerland; ^7^Agriculture and Agri-Food Canada, London, ON, Canada; ^8^Department of Biology, University of Western Ontario, London, ON, Canada; ^9^Swiss Centre for Applied Ecotoxicology (Ecotox Centre), Lausanne, Switzerland

**Keywords:** benthic communities, microbial ecotoxicology, metals, organic matter, PCB, PAH, resistance genes, urban contamination

## Abstract

Lake sediments are natural receptors for a wide range of anthropogenic contaminants including organic matter and toxicants such as trace metals, polycyclic aromatic hydrocarbons, polychlorinated biphenyls that accumulate over time. This contamination can impact benthic communities, including microorganisms which play a crucial role in biogeochemical cycling and food-webs. The present survey aimed at exploring whether anthropogenic contamination, at a large lake scale, can influence the diversity, structure and functions of microbial communities associated to surface sediment, as well as their genetic potential for resistance to metals and antibiotics. Changes in the characteristics of these communities were assessed in surface sediments collected in Lake Geneva from eight sampling sites in October 2017 and May 2018. These sampling sites were characterized by a large concentration range of metal and organic compound contamination. Variation between the two sampling periods were very limited for all sampling sites and measured microbial parameters. In contrast, spatial variations were observed, with two sites being distinct from each other, and from the other six sites. Benthic communities from the most contaminated sampling site (Vidy Bay, near the city of Lausanne) were characterized by the lowest bacterial and archaeal diversity, a distinct community composition, the highest abundance of antibiotic resistance genes and functional (respiration, denitrification, methanogenesis, phosphatase, and beta-glucosidase) activity levels. The second sampling site which is highly influenced by inputs from the Rhône River, exhibited low levels of diversity, a distinct community composition, high abundance of antibiotic resistance genes and the highest bacterial abundance. Overall, our results suggest that local anthropogenic contamination, including organic matter and toxicants, is a major driver of the diversity and functioning of sediment-microbial communities in Lake Geneva. This highlights the need to consider benthic microbial communities and a suite of complementary ecotoxicological endpoints for more effective environmental risk assessments of contaminants in lake sediments.

## Introduction

The sediment compartment in lakes can act as a sink for contaminants, including not only nutrients and organic matter but also metals, persistent organic pollutants [e.g., polycyclic aromatic hydrocarbons, polycyclic aromatic hydrocarbons (PAHs); polychlorinated biphenyls, polychlorinated biphenyls (PCBs)] and other substances such as pesticides or pollutants of emerging concerns (e.g., pharmaceuticals including antibiotics) (Xu et al., 2017; Codling et al., 2018; Golovko et al., 2020). Due to their hydrophobic nature, once deposited most of these contaminants associate strongly to the sediment (Sedláček et al., 2020). Therefore, the vertical depth profiles of contaminants in sediment cores generally reflect the temporal history of contamination, with increasing depth being associated with older contamination events (Lécrivain et al., 2018). On the other hand, horizontal gradients of contamination depend mainly on the distance from the contamination sources. The contamination of surface sediments (0–5 cm layer) in the littoral and pelagic zones of lakes can thus exhibit substantial spatial heterogeneity according to the distribution of point- and diffuse sources of pollution in the surrounding watershed, and contaminant fluxes carried into the lake by tributaries (Marvin et al., 2004; Lécrivain et al., 2019). Therefore, benthic communities in lake sediments can experience different exposures to contaminants including nutrients and organic matter according to their location. However, the resulting effects on these communities still under-investigated (Pesce et al., 2018) and little is known about the potential relationships between the spatial contamination heterogeneity in lake surface sediment, and the consequent spatial alterations of benthic community diversity, structure and functions (Haller et al., 2011; Sauvain et al., 2014; Jin et al., 2019).

In lake surface sediments, microorganisms are highly abundant and characterized by a large taxonomic and functional diversity, making them key players in a multitude of ecosystem processes (Schallenberg and Kalff, 1993; Pearman et al., 2020). These processes include biogeochemical cycling of nitrogen, the recycling of autochthonous and allochthonous organic matter, and the dissipation of organic contaminants (Vadeboncoeur et al., 2002; Haglund et al., 2003; Bedard, 2008; Schultz and Urban, 2008). Several laboratory studies have shown that environmental concentrations of metals (Mahamoud Ahmed et al., 2018, 2020) and organic contaminants such as pesticides (Widenfalk et al., 2004, 2008), PCBs (Diepens et al., 2015) and PAHs (Zhang et al., 2014) can affect the diversity, structure and functional potential of exposed sediment microbial communities. The structural and functional properties of microbial communities in lake sediments can vary horizontally at a relatively limited geographical scale, according to several local environmental factors (Krzmarzick et al., 2013; Pearman et al., 2020). However, the role of anthropogenic contamination in the spatial structuration of those microbial assemblages and the resulting effects on the functional properties of lake benthic ecosystems remain largely unknown.

The contamination of Lake Geneva has been monitored with a focus on various groups of contaminants such as PAHs, PCBs, metals, pharmaceuticals, and pesticides in surface water and sediments (e.g., Poté et al., 2008; Perazzolo et al., 2010; Hoerger et al., 2014; Larras et al., 2016). For example, a large sampling survey undertaken in 2015 mapped the contamination of the surface sediments at the whole lake scale (Loizeau et al., 2017). Sediments in the Vidy Bay near the city of Lausanne were the most contaminated by metals, organic pollutants and organic matter (Loizeau et al., 2017). The high contamination of sediments in this area has been documented for decades (Grandjean et al., 1990; Poté et al., 2008; Loizeau et al., 2013; Gascon Diez et al., 2014; Masson and Tercier-Waeber, 2014). This is mainly due to historic inputs from urban sewage effluents (Gascón Díez et al., 2017), which is also reflected by the prevalence of fecal-indicator bacteria (Poté et al., 2008) and antibiotic-resistance genes (Czekalski et al., 2014). The contamination has been suggested as an important driver of microbial community structure (Haller et al., 2011). Besides this hotspot of contamination, there is relative spatial heterogeneity in the contamination at the whole Lake scale with a strong influence of the Rhone River, which is the main Lake tributary, and of the lakeshore (Loizeau et al., 2017).

In this context, we performed two sampling campaigns to investigate the spatio-temporal variations in benthic microbial communities collected in surface sediments from eight sampling sites in Lake Geneva. These sites, which are widely geographically distributed at the lake scale, cover a large range of contamination levels by metals and organic toxicants (Loizeau et al., 2017). We included one site located in the Vidy Bay and one site located near the Rhone River mouth into the lake. The objective was to assess whether spatial and/or temporal changes in the structural and functional characteristics of the studied community are driven by anthropogenic contamination. For this purpose, microbial communities in sediment samples were characterized for bacterial and archaeal population diversity, composition and structure, bacterial abundance, functional (respiration, denitrification, methanogenesis, leucine aminopeptidase, phosphatase, and beta-glucosidase) potential activities and the abundance of gene targets associated with resistance to selected metals and antibiotics. We expected the microbial community structure from these eight sites to exhibit spatial and temporal dynamics, with functional properties linked both to community composition and to the environmental conditions prevailing in the sediment compartment on the sampling dates. Contamination levels by metals and organic toxicants were expected to select for more tolerant populations at the sampling site scale, leading to change in community structure and to increasing genetic potential of resistance, with possible impacts on ecosystem functions.

## Materials and Methods

### Sampling Sites and Sample Collection

Lake Geneva is the largest natural lake in western Europe (580 km^2^, 72 km long and 14 km wide), with a maximum depth of 309 m, and a watershed surface area of 7,999 km^2^. The lake is located between France and Switzerland, and its major tributary and outflow is the Rhône River. The water residence time is 11.3 years (CIPEL, 2018). Lake Geneva is a deep monomictic lake with irregular complete winter overturns occurring on average every 5 years (Schwefel et al., 2016).

**FIGURE 1:**
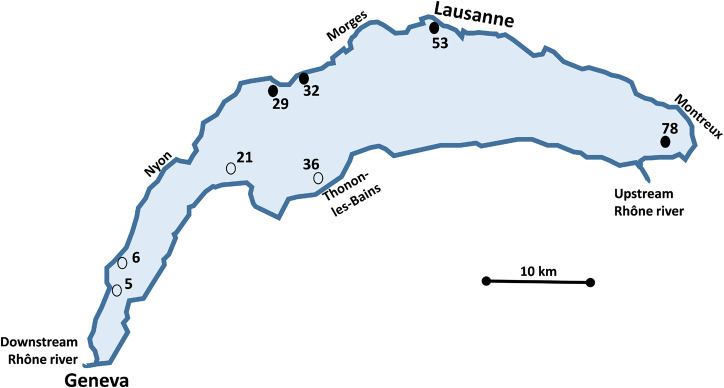
Map showing the location of Lake Geneva and of the sampling sites. ○ Sites sampled in May 2018 only; ● Sites sampled in both October 2017 and May 2018.

Sample collection was carried out on October 2017 (26th) and May 2018 (23rd, 24th, and 25th) at eight sampling sites. Sampling sites were referred as sites number 5, 6, 21, 29, 32, 36, 53, and 78 (Figure 1), according to the map of sampling sites of Loizeau et al. (2017). The sites were chosen to cover a large range of contamination levels by metals and organic toxicants, as reported by Loizeau et al. (2017). Sites 29, 32, 53, and 78 were sampled both years whereas sites 5, 6, 21, and 36 were only sampled in 2018 due to weather conditions in 2017. Sampling site descriptions, sampling date, geographical coordinates, and depth are provided in Table 1.

**TABLE 1:** Presentations of the sampling site characteristics: sampling date (in bold, those when the presented physico-chemical parameters were measured), geographical coordinates, depth, and physico-chemical sediment characteristics: organic matter (OM) content (± SD), carbonate content (± SD), total organic carbon (TOC) content, total phosphorus (Ptot), and Ti concentrations, median grain size (± SD), and the sums of sediment concentrations of the following trace metals (Cr, Co, Ni, Cu, Zn, As, Mo, Ag, Cd, Sn, Pb, and Hg), of the seven indicator PCBs, and of PAHs.

Site	Date	Coordinates		Depth	Organic matter	Carbonate	TOC	Ptot	Median grain size	Ti	Σ other metals	Σ7PCBi	ΣPAH
		Longitude	Latitude	(m)	± SD (%)	± SD (%)	(%)	(mg kg^–1^ dw)	± SD (μm)	(mg kg^–1^ dw)	(mg kg^–1^ dw)	(μg kg^–1^ dw)	(μg kg^–1^ dw)
5	**May 18**	E6.18008	N46.26927	47	8.64 ± 0.35	48.14 ± 0.83	4.35	848.6	14.77 ± 3.26	37.3 ± 1.0	206.6	5.47	1120.8
6	**May 18**	E6.19270	N46.29642	55	8.95 ± 0.10	44.33 ± 0.26	4.59	834.8	7.73 ± 2.73	37.6 ± 1.5	294.5	10.53	1372.4
21	**May 18**	E6.33359	N46.37907	50	7.18 ± 0.33	42.09 ± 0.76	2.93	750.4	7.98 ± 2.79	75 ± 1.6	267.1	9.81	1139.4
29	May 18, **Oct 17**	E6.38462	N46.45147	95	6.38 ± 0.13	31.21 ± 0.22	2.76	872.0	10.57 ± 3.01	405.5 ± 1.8	321.8	7.40	1922.2
32	May 18, **Oct 17**	E6.42316	N46.46618	22	5.27 ± 0.21	36.610.36	2.08	670.1	14.95 ± 3.90	177.7 ± 4.5	212.1	7.69	2262.5
36	**May 18**	E6.45097	N. 46.37102	32	4.79 ± 0.04	48.87 ± 0.35	2.67	549.3	19.07 ± 5.31	42.5 ± 2.4	169.8	5.20	13581.1
53	May 18, **Oct 17**	E6.58810	N46.51014	44	13.65 ± 0.09	19.70 ± 0.23	6.96	3900.0	34.82 ± 5.00	170.7 ± 6.2	1193.4	386.55	25261.9
78	May 18, **Oct 17**	E6.89261	N46.40998	60	2.73 ± 0.30	14.40 ± 0.32	0.68	659.4	11.12 ± 2.52	1735.6 ± 30.2	293.0	0.89	774.3

*Detailed metals, PCB and HAP concentrations are presented in Supplementary Tables 1–3.*

Surface sediment sampling was carried out using an Eckman grab sampler, and the top 15–25 cm of sediment were grabbed. Samples for subsequent analyses were collected in the Eckman grab, from the first 2-cm of sediment directly in the field, and stored for further analyses. For each site, three independent samples were collected. Each sample was sub-sampled directly on the boat, and samples were stored at 4°C during transport to the laboratory with arrival within 6 h.

### Sediment Chemical Characterization

The analyses of the physico-chemical properties of sediment from sites 29, 32, 53, and 78 were carried out on samples collected on October 2017, and from sites 5, 6, 21, and 36 on samples collected on May 2018. These analyses included concentrations of trace metals (Ti, Cr, Co, Ni, Cu, Zn, As, Mo, Ag, Cd, Sn, Pb, and Hg), 12 PCBs, 16 PAHs, and ancillary parameters [grain size distribution, organic matter (OM), carbonate and total organic carbon content (TOC), total phosphorus content (Ptot)]. Ti was used as a proxy of the Rhône River influence on Lake Geneva, as suspended particle load from this river mainly originate from the erosion of Alpine rocks richer in Ti-bearing minerals than rocks from the calcareous Jura mountains drained by the others tributaries, or calcite particles directly precipitated from the water column.

Sediment grain-size distribution was determined on wet sediments using a laser diffraction analyzer (Coulter LS-100, Beckman-Coulter, United States), following the procedure described by Loizeau et al. (1994).

Samples were freeze-dried in a CHRIST BETA 1–8 K freeze drying unit (−54°C, 6 Pa) for a minimum of 48 h. Organic matter and calcium carbonate (CaCO_3_) contents in sediments were estimated by Loss on Ignition. Samples were heated to 550°C for 30 min to estimate the OM mass loss and then heated to 1000°C for another 30 min to estimate the CaCO_3_ content (Dean, 1974). The CaCO_3_ content was calculated by multiplying the mass loss at 1000°C by 2.2742, the molar mass ratio of calcite to carbon dioxide.

Total phosphorus (TP) was measured with a spectrophotometer (Helios Gamma UV–Vis Thermo Electroporation, Thermo scientific, United States) at 850 nm. The sediment sample preparation for TP analysis was performed as follow; 50 mg of ignited sediment (after heating at 550°C for 1 h) were diluted in 5 mL HCl 1 N and introduced in centrifuge tubes. The mixture was ultrasonicated (at ambient temperature) during 16 h and centrifuged (4000 × rpm) during 20 min. The TP concentration was determined by measuring the absorbance of the blue complex obtained after reduction of molybdophosphoric acid according to previously described method (Murphy and Riley, 1962; Harwood et al., 1969; Burrus et al., 1990).

Quadrupole based inductively coupled plasma mass spectrometry (ICPMS, model 7700 series, Agilent) was performed following sediment digestion in Teflon bombs heated to 150°C in analytical grade 2 M HNO_3_ according to the Swiss Soil Protection Ordinance (OSol, Office Fédéral de l’environnement [OFEV], 1998). Multi-element standard solutions of different concentrations (0, 0.02, 1, 5, 20, 100, and 200 mg L^–1^) were used for calibration. The total variation coefficients for triplicate sample measurements were less than 10%.

Total Hg was analyzed by Cold Vapor Atomic Absorption 187 Spectrophotometry (CV-AAS) using an Advance Mercury Analyzer (Model AMA 254, Altec, Czech Republic) through dry mineralization and pre-concentration of Hg and amalgamation on a gold trap (Szakova et al., 2004). The detection limit and working range were 0.01 ng and 0.05–600 ng, respectively. The relative error was usually ±5% and always less than 10% (Roos-Barraclough et al., 2002).

Polychlorinated biphenyls and PAHs were quantified on freeze-dried sediments. PCBs and PAHs were characterized according to the method described in Mwanamoki et al. (2014). Briefly, 5 g of dry sediment were extracted with a mixture of acetone (20% of the volume) and hexane (80% of the volume) for 4 h with a Soxhlet apparatus. At this point, interfering sulfur compounds were removed by adding activated copper to the extract. The organic extract was then concentrated to 1 mL in a vacuum rotary evaporator. The extract was further subject to fractionation and clean-up over a chromatographic column containing 3 g of Silica gel. Three separated fractions were collected: first with 16 mL of hexane, then 35 mL of hexane, and finally 50 mL of hexane:dichloromethane (v/v, 1:1). The first fraction should contain PCBs while PAHs are distributed in the three fractions. Following reduction of the volume, chemicals were measured by gas chromatography with triple mass spectrometry detection (GC–MS/MS, Thermo Scientific, TSQ Quantum XLS Ultra, Waltham, MA, United States).

### Bacterial Abundance and Functional Potential of the Microbial Community

Bacterial abundance was estimated by flow cytometry as described in Frossard et al. (2012). Briefly, 3 g of wet sediment were suspended in 10 mL of 2% formalin containing 0.1% pyrophosphate. After an ultrasonic treatment for 1 min (Branson Digital Sonifier 250, Germany) and homogenization, 1 mL subsample of each suspension was placed on top of 0.5 mL Histodenz solution (Sigma-Aldrich, Buchs, Switzerland) and centrifuged at 17,135 × *g* at 4°C for 90 min. The upper layer, containing bacterial cells, was recovered and stained with 0.1 mL mL^–1^ of SYBER^®^ Green I (Promega, Switzerland) in anhydrous dimethylsulfoxide and incubated during 15 min in the dark. A known concentration of fluorescent beads (Beckman Coulter, Switzerland) was added to the samples as a standard to determine the cell concentration. Samples were analysed with a Gallios flow cytometer (Beckman Coulter, Switzerland).

The functional potential of the microbial community was assessed through the measurement of three enzymatic activities: ß-glucosidase, ß-glu, EC 3.2.1.21; leucine aminopeptidase, LAP, EC 3.4.11.1; and phosphatase, Pase, EC 3.1.3.1; and three metabolic activities: aerobic respiration, denitrification and methanogenesis.

Briefly, to measure enzymatic activities, 1.2 g of wet sediment were incubated for 30 min with pre-determined optimal concentrations of the fluorogenic substrates 4-methylumbelliferyl-β-D-glucopyranoside (MUF-Glu, CAS No. 18997-57-4) for β-glu, L-leucine-7-amido-4-methylcoumarin hydrochloride (Leu-AMC, CAS No. 62480-44-8) for LAP, and 4-methylumbelliferyl phosphatase (MUF-P, CAS No. 3368-04-5) for Pase (Mahamoud Ahmed et al., 2018). After the activities were stopped with glycine buffer (0.05 M glycine, 0.2 M NH_4_OH, pH 10.4), the samples were centrifuged and the fluorescence in the supernatant was measured using a microplate reader (Exc: 360 nm/Em: 460 nm, Synergy HT BioTek Instruments). Results are expressed as nmol of fluorochrome (MUF or MCA) per g of sediment dw^–1^ h^–1^.

Potential rates of aerobic respiration, denitrification and methanogenesis were measured with 10 g of wet sediment according to the protocols described by Foulquier et al. (2013) and slightly adapted by Mahamoud Ahmed et al. (2018). Sediment was mixed with 10 mL of distilled water under aerobic conditions (aerobic respiration), or 10 mL of a KNO_3_ (2.16 g L^–1^) solution under anaerobic conditions (denitrification), or 10 mL of distilled water under anaerobic conditions (methanogenesis) in 150-mL glass flasks with rubber stoppers. Incubation flasks assigned to denitrification and methanogenesis measurements were purged three times with He to achieve anaerobiosis, and the internal pressure was then adjusted to atmospheric. For denitrification measurements, 15 mL of acetylene (C_2_H_2_, 10% v/v final volume) was added to inhibit N_2_O reductase. All samples were incubated at 20°C in the dark with gentle shaking. After 2 h and 5 h, headspace gasses were sampled and analyzed by gas chromatography (Agilent 490 MICRO GC). Aerobic respiration, denitrification and methanogenesis activities are expressed as ng of gaseous product (CO_2_ or N_2_O or CH_4_, respectively) per g of sediment dw^–1^ h^–1^.

### Microbial Community Structure

#### DNA Extraction

Microbial sediment DNA was extracted from 0.5 g of wet sediment using a NucleoSpin Soil Kit (Macherey-Nagel EURL) following the manufacturer’s instructions with SL1 lysis buffer and additive Enhancer SX buffer. The extracted DNA was quantified fluorometrically after staining with QuantiFluor dsDNA Dye (QuantiFluor dsDNA System, Promega) using a Plate Chameleon^TM^ fluorometer (Hidex; excitation 485 nm, emission 590 nm).

#### High-Throughput 16S rRNA Gene Sequencing and Bioinformatic Analysis

PCR amplification for high-throughput 16S rRNA gene amplicon sequencing was carried out with the universal primer pair 515F and 909R targeting the V4–V5 hypervariable region of the 16S rRNA gene (Wang and Qian, 2009). Indexes were integrated to both primers following the dual-indexing procedure described by Kozich et al. (2013). Triplicate PCR amplification for each sample was carried out with a total amount of ∼5 ng of DNA per reaction. Amplicon products were quantified using the Picogreen assay (Life Technologies, Carlsbad, United States) and pooled equimolarly. The final pool was purified with CleanPCR beads (CleanNA). Sequencing was done by Fasteris (Geneva, Switzerland) on an Illumina HiSeq system with 2 × 300 bp. The analysis yielded 3.7 Gb of sequences with an average error rate of 1.480%, and average Q30 of 85.0%. Adapters were removed using Trimmomatic (Bolger et al., 2014) and barcodes sorted using a Fasteris internal script. Sequences were then processed using the FROGS (Find Rapidly OTUs with Galaxy Solution) Galaxy-supported pipeline (Escudié et al., 2018). Paired-end reads were joined using FLASH (Magoè and Salzberg, 2011) and a quality check was performed using FastQC. Sequences with primers having no mismatch were kept. They were then filtered by size (350–500 bp) and those containing N bases were discarded. The 16S rRNA gene sequences were then denoised and clustered using the Swarm method (Mahé et al., 2015) with a three-base maximum difference, deletion of clusters with less than 0.005% abundance and cluster occurrence in a minimum of two samples of the total library. Chimeras were removed using vchime of vsearch package (Rognes et al., 2016). Affiliation was done using the Silva SSU database 123 (Quast et al., 2013) through BLAST (Altschul et al., 1990) with allowed multiple affiliation and manual curation. All analyses were done on the Galaxy instance of the INRA MIGALE bioinformatics platform^1^. Sequences are available at GenBank under accession number PRJNA742879.

#### Microbial Community Genetic Resistance Potential

Real-time PCR quantification of bacterial 16S rRNA gene abundance was carried out using the primers 968F and 1401R according to Cébron et al. (2008). For the quantification of *cus*A (encoding a copper efflux system inner membrane protein), the primer pair cusF1 and cusR2 (Mahamoud Ahmed et al., 2020) was used, and for the quantification of *czcA* (inner membrane protein contributing to cobalt, zinc, and cadmium efflux) the primer pair czcF3 (5′-CCCTGGACTTCGGCATCATYGTBGAYGG-3′) and czcR1 (5′-GGCCATGGGGTGGAACATYTTNCC-3′) was used. The previously described reactions and cycling conditions (Mahamoud Ahmed et al., 2020) were applied to both genes. To construct standard plasmids, *czcA* and *cusA* fragments were amplified from *Burkholderia* sp. S9I1 (accession no. KP081480) and *Cupriavidus metallidurans* CH34, respectively, and cloned into the pGEM-T vector. Gene copy numbers were established using standard curves prepared with 10-fold serial dilutions of standard plasmids. All runs were performed in an iCycler iQ system (Bio-Rad) associated with iCycler Optical System Interface software (version 2.3, Bio-Rad).

The abundance of selected antibiotic resistance gene targets (*sul*1, *str*A, *str*B, *erm*B, *erm*F, *aad*A, and *bla*_OXA__–__2__0_) and the class 1 integron (*intI*1) in sediment DNA was quantified by qPCR with SYBR green or Taqman probe chemistry. All procedures, primers, quality control and standards for quantification were exactly as described in Lau et al. (2020). The identities of the quantified gene targets were verified on the basis of hybridization when using TaqMan chemistry, or melting behavior when using SYBR green. Gene abundances were expressed as a ratio with the total 16S rRNA gene copies.

### Data Analyses

All statistical analyses were performed with the R free software (version 3.4.3, R Core Team, 2018). After confirming normality of the residuals (Shapiro-Wilk test; Royston, 1982) and data homoscedasticity (Fligner-Killeen test; Conover et al., 1981), significant differences between sites and dates in the enzymatic activities, metabolic potentials, bacterial abundance, OTU richness and Shannon diversity index and genetic resistance potential were sought by analysis of variance (ANOVA) and further analyzed with a *post hoc* Tukey test. Differences were considered statistically significant when the *p*-value was below 0.05.

For bacteria and archaeal diversity, Bray-Curtis similarities (BC) between samples were calculated, and distances between samples were represented using a Principal Coordinates Analysis (PCoA) with R software (Vegan package). Functional-based community parameters (enzymatic and metabolic activities, metals and antibiotic resistance genes) were analyzed using a Principal Component Analysis (PCA). The significance of the sample groupings on the ordinations was tested using ANalysis Of SIMilarity (ANOSIM). The relationship between community composition and functional-based structures (based on PCoA and PCA 2-dimension projections, respectively) were evaluated using a Procrustean analysis (using R software, Vegan package). Procrustean analyses superimpose, scale and rotate one data matrix upon the other until an optimal fit is found (Jackson and Harvey, 1993; Peres-Neto and Jackson, 2001; Heino et al., 2004). Estimated residuals between original values and the derived best fit solution give the m12 statistic, with a low m12 statistic indicating a good level of correspondence between data matrices (Paavola et al., 2006). The PROTEST permutation procedure (999 permutations) was used to assess the statistical significance of the Procrustean fit between the two matrices (Peres-Neto and Jackson, 2001; Paavola et al., 2006). Vectors of environmental variable data were fitted onto the procrustean analysis plot using the envfit function of the vegan package. This function calculates the goodness-of-fit values (R^2^) for environmental variables onto the procrustean analysis plot and the significance of each correlation was tested based on 999 random permutations (Oksanen et al., 2020).

## Results

### Environmental Conditions and Sediment Contamination at the Sampling Sites

The main sampling site characteristics are presented in Table 1. Surface sediments were collected at a sampling depth ranging from 22 m (site 32) to 95 m (site 29), and the median grain size varied from 8 μm (sites 6 and 21) to 35 μm (site 53). Sediments from the sites 53 and 78 had the highest and lowest organic matter content (13.6 and 2.7%, respectively) and total organic carbon (7.0 and 0.7%, respectively). These two sites were also characterized by lower carbonate content (about 14.4 and 19.7%, respectively) than the six other sites (i.e., 5, 6, 21, 29, 32, and 36) where it varied between 31.2% (site 29) and 48.9% (site 36). Total phosphorus concentrations were 4.5- to 7-fold higher in the site 53 (3900 mg kg^–1^ dw) than in the seven other sites (659 to 849 mg kg^–1^ dw). Sediments from the site 53 contained high total concentrations of metals (about 1193 mg kg^–1^ dw) compared to the seven other ones, where it varied from 170 mg kg^–1^ dw (site 36) to 322 mg kg^–1^ dw (site 29). The high total concentration of metals in the site 53 was mainly due to zinc and copper (Zn and Cu, 645 and 281 mg kg^–1^ dw, respectively; Supplementary Table 1). The sediments from site 78 contained high concentrations of titanium (Ti; 1736 mg kg^–1^ dw) while for the other sites Ti concentrations ranged between 37 and 405 mg kg^–1^ dw. The site 53 was also characterized by higher values for the sum of the seven indicator PCB (Σ7PCBi) (387 μg Σ7PCBi kg^–1^ dw): more than 400-fold higher than those observed in the site 78 (0.9 μg Σ7PCBi kg^–1^ dw) and 36- to 75-fold higher than those observed in the six other sites (5.2–10.5 μg Σ7PCBi kg^–1^ dw) (Supplementary Table 2). Sediments from sites 53 and 36 contained higher concentrations of PAH (25262 μg kg^–1^ dw and 13581 μg kg^–1^ dw, respectively) compared to the six other ones, where it varied from 774 μg kg^–1^ dw (site 78) to 2263 μg kg^–1^ dw (site 32) (Supplementary Table 3).

### Functional Potential

**FIGURE 2:**
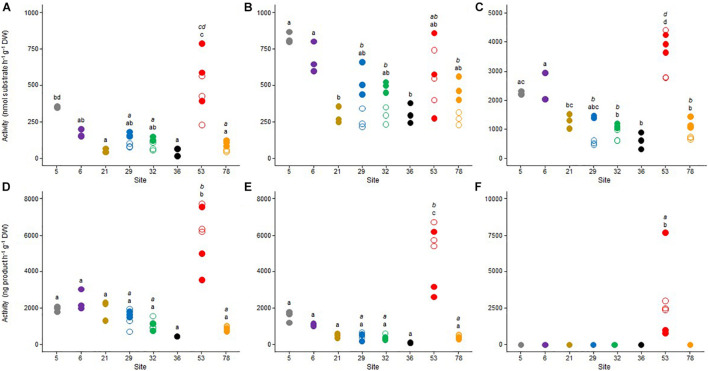
Dot plots of the values obtained for **(A)** beta-glucosidase, **(B)** leucine-aminopeptidase, **(C)** phosphatase, **(D)** respiration, **(E)** denitrification, and **(F)** methanogenesis benthic microbial activities for the triplicate samples from the 8 studied sites in Lake Geneva in October 2017 (empty symbols) and May 2018 (plain symbols). For each sampling site (and date if applicable – October 2017, italic letter and May 2018, roman letter), different letters indicate significant differences between treatments (*P* < 0.05, Tukey test).

A set of potential microbial activities involved in the C cycle (ß-glu, respiration, and methanogenesis), the N cycle (LAP and denitrification) and the P cycle (Pase) was measured (Figure 2). The results reflected a high spatial heterogeneity between the sampling sites. With the exception of the LAP activity (Figure 2B), sediment microbial communities from the site 53 exhibited the highest functional potential, both in October 2017 and May 2018. Methanogenesis activity (Figure 2F) was only detected in this site and ß-glu (Figure 2A), Pase (Figure 2C), aerobic respiration (Figure 2D) and denitrification (Figure 2E) were also significantly higher than in the other sampling sites (*p* < 0.05). Significant but less marked spatial differences were also observed between the seven other sites (i.e., 5, 6, 21, 29, 32, 36, and 78) for the three enzymatic activities, which were generally the highest in sites 5 and 6 and the lowest in sites 21 and 36 (Figures 2A–C). In contrast, no significant difference was observed between these seven sites for both aerobic respiration (Figure 2D) and denitrification (Figure 2E).

Seasonal variations between the two sampling periods were less pronounced in the four sites that have been sampled in 2017 and 2018 (i.e., 29, 32, 53, and 78). Significant differences (*p* < 0.05) between October 2017 and May 2018 were only observed in site 53 for denitrification (Figure 2D) and methanogenesis (Figure 2E), with the highest values measured in October 2017.

### Microbial Community Structure

**FIGURE 3:**
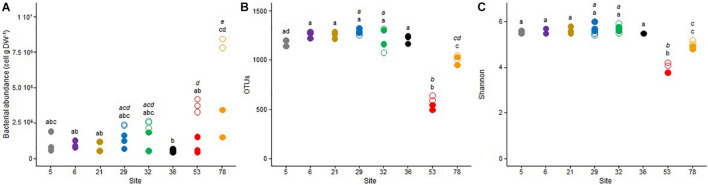
Dot plots of the **(A)** bacterial abundance, **(B)** observed OTU richness, and **(C)** Shannon diversity index for the triplicate samples from the 8 studied sites in Lake Geneva in October 2017 (open symbols) and May 2018 (closed symbols). For each sampling site (and date if applicable – October 2017, italic letter and May 2018, roman letter), different letters indicate significant differences between treatments (*P* < 0.05, Tukey test).

The mean bacterial abundance in the sample sediments ranged between 0.55 × 10^6^ to 8.03 × 10^6^ cells g^–1^ dw (Figure 3A). A high spatial variability was observed in October 2017, when the bacterial community from site 78 exhibited the highest abundance (about 8.0 × 10^6^ cells g^–1^ dw), which was about twice higher than the one measured in site 53, and threefold higher than those measured in sites 29 and 32. In these four sites, the bacterial abundance was reduced by about two- to fourfold between October 2017 and May 2018. In May 2018, abundances were more stable between the different sites, ranging from 0.55 × 10^6^ at site 36 to 2.80 × 10^6^ cells g^–1^ dw at site 78.

High-throughput amplicon sequencing of the 16S rRNA gene revealed no temporal difference in the number of OTUs affiliated to the archaea and bacteria domains (Figure 3B) and the resulting Shannon-Wiener diversity index (Figure 3C) between October 2017 and May 2018. Based on these two parameters, communities from site 53, and to a lesser extent, from site 78, exhibited the lowest diversity, whereas these two parameters were relatively similar between the six other sites. According to the sampling date and the sample replicate, the number of OTUs varied from 503 to 626 in site 53, from 947 to 1048 in site 78, and from 1067 to 1311 in the six other sites. The mean Shannon-Wiener diversity index was close to 4.0 (±0.2) in site 53, 4.9 (±0.1) in site 78, and between 5.4 and 5.8 in the six other sites.

**FIGURE 4:**
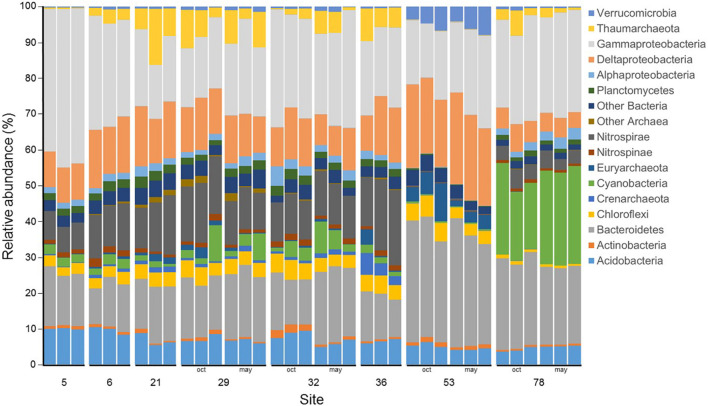
Microbial community composition for the triplicate samples from the 8 studied sites in Lake Geneva in October 2017 and May 2018. The 15 more abundant phylum (or class for Proteobacteria) are represented and the remaining phylum (or class for Proteobacteria) are grouped under “Other Archaea” or “Other Bacteria”. Data is presented are relative abundance of the read counts.

At the Phylum (or Class for Proteobacteria) level, the microbial community was dominated by members of the Gammaproteobacteria (25.2 ± 7.0% of the reads), followed by members of the Bacteroidetes (19.4 ± 6.9%), Deltaproteobacteria (13.1 ± 5.4%), Nitrospirae (9.3 ± 5.9%), and Acidobacteria (6.8 ± 1.9%) (Figure 4). Community composition at site 53 was especially dominated by members of the Bacteroidetes (32%), Deltaproteobacteria (22%) and Gammaproteobacteria (20%), whereas at site 78, community composition was dominated by members of the Gammaproteobacteria (27%), Cyanobacteria (24%) and Bacteroidetes (23%). Site 5 community composition was characterized by the most important proportion of members of the Gammaproteobacteria (42%). Members of the Nitrospirae were present at low abundances at sites 53 (0.30%), 78 (4.23%) and 5 (8.05%), whereas they ranged between 11% (sites 29 and 32) and 18% (site 36). Among the less abundant groups, Actinobacteria, Chloroflexi, Nitrospinae, and other Archaea and Bacteria did not exhibit any temporal or spatial trends. For the main archaeal Phylum, Crenarchaeota peaked up to 4% at site 36, but remained below 1.5% at other sites, Euryarchaeota were generally below 2% except at site 53 where they represented 7% of the community in October 2017 and 3% in May 2018, and Thaumarchaeota represented less than 0.5% of the community at sites 5 and 53 but between 2 and 10% elsewhere. Finally, Verrumicrobia were below 1% in every samples but at site 53 where they accounted for 5 and 6% of the community in October 2017 and May 2018, respectively.

### Metal and Antibiotic Resistance Potential

**FIGURE 5:**
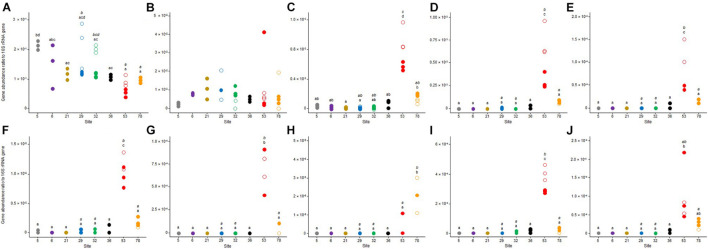
Dotplots of the gene abundance ratio to 16S rRNA gene abundance of **(A)** *cus*A, **(B)** *czc*A, **(C)** *sul*1, **(D)** *str*A, **(E)** *str*B, **(F)** *intI*1, **(G)** *erm*B, **(H)** *erm*F, **(I)** *aad*A, and **(J)** *bla*_*OX*__A__–__2__0_ genes for the triplicate samples from the eight sites in Lake Geneva in October 2017 (open symbols) and May 2018 (closed symbols). For each sampling site (and date if applicable – October 2017, italic letter and May 2018, roman letter), different letters indicate significant differences between treatments (*P* < 0.05, Tukey test).

Two distinct sets of quantitative PCRs were performed to, respectively, quantify two genes involved in microbial resistance to metals (*cusA* and *czcA*, Figures 5A,B) and eight genes conferring antibiotic resistance (*sul1*, *strA*, *strB*, *ermB*, *ermF*, *aadA*, and *bla*_*OXA*__20_), and the class 1 integron integrase *intI1* (Figures 5C–J).

Both metal resistance genes were detected in every site and the two sampling dates (Figures 5A,B). The *cusA* gene had the highest relative abundance with mean *cusA* to 16S rRNA gene relative abundances varying between about 0.49 × 10^–3^ to 210 × 10^–3^ depending on the sampling site and date (Figure 5A). No significant temporal variation was observed for this gene between the two sampling dates with the exception of sites 29 and 32 where mean values were about twofold higher (*p* < 0.05) in October 2017 than in May 2018. Spatial differences among the sites varied according to the sampling date. In October 2017, the relative abundance of *cusA* was significantly higher in sites 29 and 32 than in sites 53 and 78 (*p* < 0.05). In May 2018, the highest relative abundance was observed in site 5 (204 × 10^–3^ ± 14 × 10^–3^) with values significantly higher than those recorded in sites 21, 32, 36, 53, and 78 (*p* < 0.05). The mean relative abundances of *czcA* to 16S rRNA varied between 1.42 × 10^–3^ and 15.3 × 10^–3^ with relatively high variations among replicates and no significant difference between sampling sites or between sampling dates (Figure 5B).

The eight genes for antibiotic resistance (Figures 5C–J) were less represented than *cusA* and *czcA*. The class 1 integron marker *intI1* was detected in all samples with the highest relative abundances with mean values varying between 98.7 × 10^–6^ and 11.2 × 10^–3^ (Figure 5F). The genes *sul1*, *strA*, and *aadA* (Figures 5C,D,I) were also detected in all the samples but their highest relative abundance value was lower than 1 × 10^–3^. The detection of *strB*, *ermB*, *ermF*, and *bla*_OX__A__–__20_ (Figures 5E,G,H,J) genes was variable according to sampling site, date and sample replicate, with mean relative abundances always lower than 1 × 10^–4^. In addition to the difference among the measured genes, the results showed a high spatial heterogeneity among the sampling sites. The general tendency showed that sediment microbial communities from site 53, and to a lesser extent those from site 78, were characterized by higher relative abundances of the eight genes than the communities from the six other sites (i.e., 5, 6, 21, 29, 32, and 36). Indeed, sediment microbial communities collected in site 53 were characterized by the highest relative abundances of seven of the eight measured genes. The only exception was for the gene *ermF*, which was about threefold more represented in site 78 than in site 53 (Figure 5H). This gene was under the detection limit in the other sampling sites. At site 53, the other seven genes relative abundances were about 3- to 28-fold more abundant than in site 78 and about 30- to 2000-fold more abundant than at the 6 other sites. Moreover, no significant difference in the relative abundance of the eight genes conferring antibiotic resistance was observed in those sites (i.e., 5, 6, 21, 29, 32, and 36) (Figures 5C–J). No temporal variation was observed between the two sampling dates with the exception of site 53 where relative abundances were significantly higher in October 2017 than in May 2018 for the genes *sul1* (Figure 5C), *strA* (Figure 5D), *strB* (Figure 5E), *int1* (Figure 5F), and *aadA* (Figure 5I).

### Environmental Drivers of Microbial Structure and Functional-Based Parameters

**FIGURE 6:**
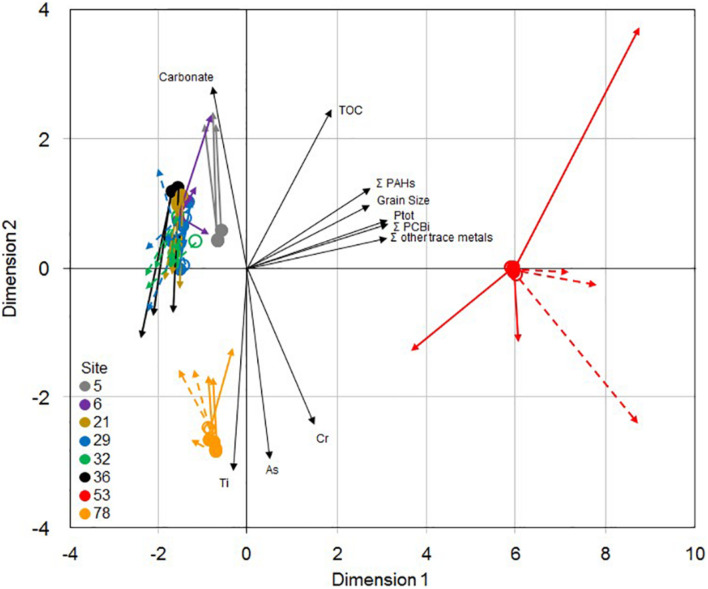
Two-dimensional representation of the procrustean analysis of 16S rRNA gene-based bacterial community structure (PCoA analysis of Bray-Curtis distances, origin of colored arrows) and functional-based community parameters (PCA analysis of microbial activities, metals and antibiotic resistance gene relative abundances, arrival of colored arrows) for the triplicate samples from the 8 studied sites in Lake Geneva in October 2017 (empty symbols) and May 2018 (plain symbols). Black arrows are environmental variables showing significant correlations with the sample 2-dimensional projections.

The 2-dimensional representation of the procrustean analysis of 16S rRNA gene-based bacterial community structure (PCoA, the first 2 axes explained 74% of the variance) and functional-based community parameters (PCA, the first 2 axes explained 76% of the variance) (Figure 6) showed that sites 53 and 78 were distinct from each other, and from the six other sites (ANOSIM; *p* < 0.0001 for PCoA, and *p* < 0.01 for PCA), with very limited differences between the two sampling dates. Among these six sites, communities from site 5 were also slightly distinct both according to their structure and their functional parameters. The phylogenetic structure and the functional and resistance pattern of the communities displayed a highly significant level of concordance (Procrustean analysis, residual sum of squares = 0.340; m12 = 0.812; *p* < 0.001), as indicated by the relatively limited length of the colored arrows on the 2-dimension projection.

The sediment physico-chemical characteristics that were correlated with the microbial community ordinations (Figure 4 and Supplementary Table 4) were organic toxicants (PCB and PAH), phosphorus concentrations and median grain size, which were higher at site 53, some metals, which were high at sites 78 (As and Cr) and 53 (sum of Co, Ni, Cu, Zn, Mo, Ag, Cd, Sn, Pb, and Hg concentrations) and carbonate, which was lower at sites 53 and 78 (Table 1). Site 78 microbial community ordination was also significantly correlated to Ti concentration.

## Discussion

This study investigated *in situ* the possible influence of the anthropogenic contamination of Lake Geneva surface sediments on benthic microbial communities by assessing spatiotemporal changes in their structure, their functional potential and their genetic potential for resistance to selected metals and antibiotics. Our results provide evidence that the multiple contaminants in sediments of Lake Geneva alter the diversity of microbial communities with consequences for the important ecological functions they provide. The magnitude of these effects depends on the types and the concentrations of contaminants in the sediments. All contaminant concentrations measured in the present study are in agreement with previous findings (Haller et al., 2011; Gascon Diez et al., 2014; Masson and Tercier-Waeber, 2014; Gascón Díez et al., 2017; Loizeau et al., 2017). In addition, our results also revealed high concentrations of PAH (>13.5 μg kg^–1^ dw) in sediments collected at site 36. Such a contamination was not reported in Loizeau et al. (2017) because organic toxicants (PAH and PCB) were not analyzed in this study site (and also not in sites 5 and 21). Our results suggest that the multi-contamination (PAH, PCB, metals, phosphorus, organic matter, and possibly microbial and genetic material) of sediments observed in the Vidy Bay (site 53) is a strong driver of sediment microbial community structure and diversity, the latter being significantly lower than that at other sites Some phyla were significantly less represented in the Vidy Bay, such as Planctomycetes, the *Nitrospira* genus from the phylum of Nitrospirae, whereas Euryarchaeota were mainly detected in this highly contaminated site. This archaeal phylum includes both the archaeal sulfide reducers as well as the methanogens (Chrencik and Marsh, 2012). Potential methanogenesis activity was only detected at this site, consistent with the observed abundance of methanogenic archeae, belonging to the *Methanobacterium*, *Methanolinea*, *Methanoregula*, *Methanosaeta*, *Methanosarcina* genera and to the *Methanomassiliicoccaceae* family.

In the present study, the Verrucomicrobia phylum was also more represented in the Vidy Bay than in the other sites, in contrast to previous findings (Haller et al., 2011). However, this is in line with other observations where this phylum was more abundant in very urbanized rivers than in suburban rivers (Lin et al., 2019). The urban contamination is also probably responsible for the observed higher relative abundance of members of the Bacteroidetes phylum, which includes bacteria from the human and animal gastrointestinal tract, frequently detected in wastewater, and generally considered as fecal indicators in the environment (Gomez-Donate et al., 2016; Su et al., 2017; Niestępski et al., 2020). In the Vidy Bay, fecal indicator bacteria and other activated sludge bacteria from local WWTP inputs were previously detected (Haller et al., 2009; Sauvain et al., 2014). Bacteroidetes are known to harbor a large variety of metal- or antibiotic-resistance genes that can favor their establishment and development in contaminated sediments (Chen et al., 2018; Niestępski et al., 2020). The higher abundance of gene markers associated with resistance to various classes of antibiotics (*sul1, strA*, *strB*, *ermB*, *ermF*, and *bla_OXA__–__20_*) and of the class 1 integron (*intI1*) detected in the Vidy Bay sediment is in accordance with the high occurrence of multiresistant bacteria and antibiotic-resistance genes previously reported at this site (Czekalski et al., 2012, 2014). Reporting in 2011 a 200-fold increase of the relative abundances of six antibiotic-resistance genes (including *sul1*) as compared to sediment sampled in the center of the lake, Czekalski et al. (2014) hypothesized that the increasing abundance of antibiotic-resistance genes was mainly due to direct inputs from wastewater effluents rather than *in situ* development of antibiotic-resistant bacteria in the lake sediment following their chronic exposure to contaminants. In the present study, given the high metal concentrations observed in the surface sediment from the Vidy Bay, the hypothesis of co-selection of antibiotic resistance by this kind of contaminant cannot be excluded (Poole, 2017; Silva et al., 2021). Although pharmaceutical residues in the collected sediments were not analyzed it is more than probable that site 53 sediment were contaminated by antibiotics (Morasch et al., 2010; Bonvin et al., 2011, 2013), potentially favoring the development of antibiotic resistance in sediment communities in response to antibiotic selection pressure (Bengtsson-Palme and Larsson, 2016). In contrast, the analysis of the metal-resistance genes *cusA* (involved in copper resistance) and *czcA* (involved in cobalt-zinc-cadmium resistance) did not show higher relative abundances in the Vidy Bay, even if this site had the highest concentrations of Cu (about 281 mg kg^–1^ dw), Zn (about 645 mg kg^–1^ dw) and Cd (about 1.5 mg kg^–1^ dw), which were 3- to 12-fold higher than the contamination with these three metals in the seven other stations. Significant increases in the relative abundance of the *cusA* and *czcA* genes in contaminated sediments compared to uncontaminated ones has been previously reported by Besaury et al. (2013) and Roosa et al. (2014). In both cases, however, the level of metal contamination was very high (total Cu > 1400 mg kg^–1^, Besaury et al., 2013; total Zn > 3000 mg kg^–1^ and Cd > 35 mg kg^–1^, Roosa et al., 2014), as was the potential metal bioavailability. Altogether, these results questions whether the qPCR quantification of these metal resistance genes is sensitive enough when contamination gradients are weak, or whether metal bioavailability in the Vidy Bay is poor and causes limited selection pressure.

More surprisingly, a notable and sometimes significant increase in the relative abundance of antibiotic-resistance genes was observed in the site 78 in comparison to the other sites other than the Vidy Bay one. This was especially the case for the *ermB* and *erm*F genes, responsible for erythromycin resistance, which were only (*erm*F) or mainly (*ermB*) detected in site 53 and 78. It cannot be excluded that the surface sediment from the site 78 has been directly contaminated by antibiotic-resistant bacteria and/or antibiotics coming from the upper section of the Rhône River, which has been shown to be an important source of contamination of Lake Geneva with pharmaceutical substances (Loizeau et al., 2013). In this site, benthic microbial richness and diversity index was impacted, being 10 to 20% lower than those observed in the other sites (excluding 53) and community composition was also affected. The relative abundance of Bacteroidetes members could also confirm inputs from sewage water located in the upper Rhône section (see related discussion above for site 53). Cyanobacteria were also significantly more represented at site 78 than the other sites, showing that this kind of microorganisms was particularly adapted to the local site characteristics. Given the water column height at this site and associated limited light penetration, it is unlikely that these microorganisms are part of a microbial mat developing at the sediment – water interface, but nutrients flowing in from the Rhône River might promote their planktonic growth following which they are deposited into sediment. Previous studies (Loizeau et al., 2012; Silva et al., 2019) found that sediment accumulation was 5- to 10-fold more rapid at this site compared to other areas of the lake.

Despite their geographical distance and their notable differences in depth and other physico-chemical characteristics, such as total organic carbon or grain size, the sediment communities collected in the less contaminated sites (i.e., 5, 6, 21, 29, and 32) and at site 36, only contaminated by high concentrations of PAH, exhibited relatively comparable structural and functional properties. All these sampling sites (as well as sites 53 and 78) had a depth below 100 m out of the 309 m of maximal depth for Lake Geneva. Extrapolation of the probe sensor data collected for the environmental survey of Lake Geneva at the deepest site indicated that water temperature at the sediment interface varied between 6 and 14°C in October 2017 and was about 6–7°C in Mai 2018, and that dissolved oxygen concentrations increased from between 6 and 8 mg L^–1^ in October 2017 and between 9 and 12 mg L^–1^ in May 2018 (Rimet et al., 2020), because of the first 201 m water column partial turnover on March 6, 2018 (CIPEL, 2019). Even if this data should be interpreted with care since it might not perfectly reflect the local conditions prevailing at sampling sites, whatever the measured parameter, no significant difference was observed between the sediment microbial communities collected in the shallowest (22 m; site 32) and deepest (95 m, site 29) sites. As revealed by the multivariate analysis (Figure 6), it confirms that depth, and indirectly related environmental parameters such as dissolved oxygen, light intensity, temperature, and nutrient availability (Böer et al., 2009), was not a major driver of benthic microbial community structure and functions in Lake Geneva, in contrast to other studies that linked microbial community structure changes with water depth (Edlund et al., 2006; Hewson et al., 2007; Zhang et al., 2014; Ding et al., 2015; Ruuskanen et al., 2018; Wu et al., 2019). The only observed differences between sites 5, 6, 21, 29, 32, and 36 concerned the enzymatic activities (ß-glu, LAP, and Pase), which were higher at site 5, and to a lesser extent, at site 6. This difference could be explained, at least partly, by a higher amount of available substrates, as suggested by the higher organic matter and total organic carbon contents measured in these samples. Based on these results, it can be argued that the contamination of the sediment with high concentrations of PAH at site 36 (>13.5 mg ΣPAH kg^–1^ dw) exerts no or very limited effects on the functional potential, the structure and the composition of the microbial communities, which were almost similar to those from sites characterized by 6- to 12-fold lower PAH concentrations. The limited effects of chronic PAH contamination on sediment microbial community diversity and functions has likewise been observed in coastal ecosystems (Hernandez-Raquet et al., 2006; Païssé et al., 2008; Zhang et al., 2008; Jeanbille et al., 2016), in contrast to the more important influence of other contaminants such as metals (Sun et al., 2012, 2013). The small effect of PAHs could be due to their limited toxicity (Païssé et al., 2010) and/or their limited bioavailability because of their ready sorption to sediments (Kraaij et al., 2002; Ghosh et al., 2003).

Despite the evident negative effects of the anthropogenic contamination on the diversity of sediment microbial communities of sites 53 and 78, no inhibition in their functional potential was observed. On the contrary, communities from site 53 exhibited the highest potential for most of the measured activities (i.e., ß-glu, Pase, aerobic respiration, denitrification and methanogenesis). It suggests that the observed shifts in their community composition reflects a long-term selection of benthic microbial species that are strongly adapted to their environment, allowing them to benefit from the regular inputs of nutrients and organic substrates that can be used as energy sources, thus making them key players in benthic biogeochemical cycles (Huang et al., 2019, Steinsberger et al., 2019). This long-term adaptation hypothesis can be supported by the very limited temporal variations that were observed in the present study between the two sampling dates. Indeed, at the four sites sampled twice (i.e., 29, 32, 53, and 78), sediment community diversity and composition were very similar in October 2017 and May 2018, as illustrated in the multivariate analysis (Figure 6). It suggests, that, the structural characteristics of the surface sediment microbial communities of Lake Geneva are relatively stable over time, unlike the case for shallower lakes (Peng et al., 2017; Wan et al., 2017; Zhang et al., 2019).

## Conclusion

Using a whole lake approach, the present study highlighted that surface sediment microbial communities were impacted by anthropogenic load of contaminants including toxicants, organic matter, nutrients, and microorganisms in wastewater discharge affecting their diversity, enzymatic and metabolic functional potential as well as their genetic potential resistance to antimicrobial agents. These forcing factors appeared to yield to a more important structuration of the microbial parameters studied than the classical environmental factors generally controlling microbial diversity in lakes.

Given the ecological role of microbial communities in lake sediments, further research to better understand the consequences of anthropogenic contamination on microbial communities should focus on larger scale approaches, including more sites to cover a finer gradient of contamination in order to distinguish the relative importance of the different type of contaminants, integrating the full range of water depths from the littoral to the deeper areas, and considering a more important depth of the sediment compartment to take into account the full range of metabolic activities carried out. Together this would improve the ecotoxicological assessment of sediments in lake ecosystems to understand the consequences of multiple stress at the community level on microbial key activities and functions.

## Data Availability Statement

The datasets presented in this study can be found in the NCBI at https://www.ncbi.nlm.nih.gov/sra/PRJNA742879.

## Author Contributions

BF and SP conceived and designed the study. EL, J-LL, BF, and SP organized and performed the sampling. EL, CB, PB, AT, and ET were responsible for the microbial analyses. J-LL, EN, and BF were responsible for the physico-chemical analyses. EL conducted the bioinformatic and biostatistical analyses. EL and SP drafted the first version of the manuscript. All co-authors analyzed and interpreted the data, contributed to subsequent revisions to the manuscript, and approved its final submitted version.

## Conflict of Interest

The authors declare that the research was conducted in the absence of any commercial or financial relationships that could be construed as a potential conflict of interest.

## Publisher’s Note

All claims expressed in this article are solely those of the authors and do not necessarily represent those of their affiliated organizations, or those of the publisher, the editors and the reviewers. Any product that may be evaluated in this article, or claim that may be made by its manufacturer, is not guaranteed or endorsed by the publisher.

## Footnotes

1
http://migale.jouy.inra.fr
