# Supplementary material for: Diversity, Functions and Antibiotic Resistance of Sediment Microbial Communities From Lake Geneva Are Driven by the Spatial Distribution of Anthropogenic Contamination
Source: Front Microbiol. 2021 Oct 18;12:738629. doi: 10.3389/fmicb.2021.738629 (PMC8560053; doi:10.3389/fmicb.2021.738629)
Supplement: Supplementary file 1 [file Table_1.DOCX]

**Diversity, functions and antibiotic resistance of sediment microbial communities from Lake Geneva are driven by the spatial distribution of anthropogenic contamination**

***Emilie Lyautey^1,2^, Chloé Bonnineau^1^, Patrick Billard^3^, Jean-Luc Loizeau^4^, Emmanuel Naffrechoux^5^, Ahmed Tlili^6^, Edward Topp^7^, Benoît J.D. Ferrari^8^, Stéphane Pesce^1^***

*^1^ INRAE UR RiverLy,* *69625 Villeurbanne, France*

*^2^ Univ. Savoie Mont Blanc, INRAE, CARRTEL, 74200 Thonon-les-Bains, France*

*^3^ Université de Lorraine, CNRS, LIEC, F-54000 Nancy, France*

*^4^ University of Geneva, Dept. F-A. Forel for environmental and aquatic sciences, Geneva, Switzerland*

*^5^ Univ. Savoie Mont Blanc, EDYTEM, 73000 Chambéry, France*

*^6^ Eawag, Swiss Federal Institute of Aquatic Science and Technology, 8600 Dübendorf, Switzerland*

*^7^ Agriculture and Agri-Food Canada, London, Ontario, Canada*

*^8^ Centre Ecotox, 1015 Lausanne, Switzerland*

***Correspondence:***

*Dr. Stéphane Pesce*

*stephane.pesce@inrae.fr*

**SUPPLEMENTARY MATERIALS**

.

**Supplementary Table 1 |** Sediment concentrations (± *SD*) of trace metals (Ti, Cr, Co, Ni, Cu, Zn, As, Mo, Ag, Cd, Sn, Pb, and Hg) at the 8 studied sites.

| **Site** | **Ti** | **Cr** | **Co** | **Ni** | **Cu** | **Zn** | **As** | **Mo** | **Ag** | **Cd** | **Sn** | **Pb** | **Hg** | |
| --- | --- | --- | --- | --- | --- | --- | --- | --- | --- | --- | --- | --- | --- | --- |
| average ± SD (mg kg^-1^ dw) | | | | | | | | | | | | | |  |
| 5 | 37.3 ± 1.0 | 31.9 ± 0.7 | 7.5 ± 0.1 | 45.4 ± 5.3 | 31.8 ± 0.7 | 79.2 ± 1.5 | 2.3 ± 0.1 |  | 0.02 ± 0.00 | 0.27 ± 0.03 | 1.08 ± 1.68 | 7.0 ± 0.4 | 0.07 ± 0.0 | |
| 6 | 37.6 ± 1.5 | 38.6 ± 1.0 | 10.5 ± 0.3 | 57.5 ± 13.3 | 45.0 ± 1.6 | 114.5 ± 3.6 | 3.2 ± 0 | 0.15 ± 0.02 | 0.07 ± 0.01 | 0.44 ± 0.05 | 0.06 ± 0.03 | 24.2 ± 2.5 | 0.19 ± 0.0 | |
| 21 | 75.0 ± 1.6 | 40.5 ± 0.9 | 10.5 ± 0.2 | 50.1 ± 1.3 | 40.2 ± 0.6 | 99.9 ± 0.9 | 3.3 ± 0.1 | 0.03 ± 0.01 | 0.16 ± 0.03 | 0.37 ± 0.07 | 0.05 ± 0.00 | 21.7 ± 3.1 | 0.25 ± 0.01 | |
| 29 | 405.5 ± 1.8 | 53.9 ± 1.2 | 12.5 ± 0.1 | 53.0 ± 0.6 | 53.1 ± 0.5 | 114.6 ± 1.1 | 5.0 ± 0.1 | 0.26 ± 0.01 | 0.33 ± 0.08 | 0.40 ± 0.08 | 0.37 ± 0.12 | 28.1 ± 4.5 | 0.15 ± 0.11 | |
| 32 | 177.7 ± 4.5 | 35.6 ± 0.9 | 8.3 ± 0.1 | 37.4 ± 0.7 | 31.6 ± 0.5 | 79.2 ± 0.8 | 2.7 ± 0.0 |  | 0.15 ± 0.02 | 0.28 ± 0.04 | 0.06 ± 0.01 | 16.5 ± 2.0 | 0.21 ± 0.03 | |
| 36 | 42.5 ± 2.4 | 22.7 ± 0.9 | 7.2 ± 0.3 | 37.1 ± 4.5 | 22.5 ± 0.8 | 60.9 ± 2.4 | 2.6 ± 0.1 |  | 0.04 ± 0.02 | 0.33 ± 0.04 | 0.05 ± 0.03 | 16.2 ± 1.4 | 0.22 ± 0.01 | |
| 53 | 170.7 ± 6.2 | 75.8 ± 2.5 | 8.3 ± 0.3 | 42.5 ± 2.0 | 280.5 ± 9.5 | 645.2 ± 68.3 | 6.8 ± 0.3 | 1.15 ± 0.06 | 3.36 ± 0.40 | 1.54 ± 0.06 | 3.54 ± 0.07 | 123.3 ± 5.6 | 1.47 ± 0.03 | |
| 78 | 1735.6 ± 30.2 | 64.7 ± 1.5 | 14.9 ± 0.2 | 52.8 ± 0.9 | 29.4 ± 0.5 | 99.7 ± 2.3 | 9.4 ± 0.2 | 0.3 ± 0.01 | 0.05 ± 0.01 | 0.25 ± 0.03 | 0.21 ± 0.07 | 21.3 ± 2.8 | 0.03 ± 0.0 | |

**Supplementary Table 2 |** Sediment concentrations of 12 PCB congeners and Σ7PCBi (sum of the 7 indicator PCB, as indicated using the sign *) at the 8 studied sites.

| **Site** | **PCB 28*** | **PCB 52*** | **PCB 101*** | **PCB 149** | **PCB 118*** | **PCB 153*** | **PCB 105** | **PCB 138*** | **PCB 128** | **PCB 156** | **PCB 180*** | **PCB 170** | **Σ7PCBi** |
| --- | --- | --- | --- | --- | --- | --- | --- | --- | --- | --- | --- | --- | --- |
|  | (µg kg^-1^ dw) | | | | | | | | | | | | |
| 5 | 0.08 | 0.21 | 0.70 | 0.96 | 0.60 | 1.70 | 0.30 | 1.46 | 0.22 | 0.15 | 1.31 | 0.60 | 5.47 |
| 6 | 0.39 | 0.54 | 1.54 | 2.00 | 1.31 | 3.48 | 0.62 | 2.81 | 0.44 | 0.24 | 1.77 | 0.81 | 10.53 |
| 21 | 0.40 | 0.52 | 1.41 | 1.91 | 1.18 | 3.14 | 0.54 | 2.68 | 0.44 | 0.20 | 1.65 | 0.79 | 9.81 |
| 29 | 0.26 | 0.43 | 1.19 | 1.65 | 0.93 | 2.63 | 0.41 | 1.85 | 0.30 | 0.19 | 1.04 | 0.52 | 7.40 |
| 32 | 0.25 | 0.49 | 1.29 | 1.42 | 0.95 | 2.41 | 0.40 | 2.09 | 0.39 | 0.22 | 1.16 | 0.59 | 7.69 |
| 36 | 0.22 | 0.26 | 0.64 | 0.83 | 0.63 | 1.75 | 0.35 | 1.40 | 0.24 | 0.11 | 0.92 | 0.33 | 5.20 |
| 53 | 37.00 | 32.33 | 59.50 | 76.66 | 35.98 | 112.87 | 14.24 | 90.33 | 13.02 | 9.16 | 54.52 | 32.91 | 386.55 |
| 78 | 0.05 | 0.06 | 0.15 | 0.17 | 0.11 | 0.28 | 0.04 | 0.23 | 0.04 | 0.02 | 0.12 | 0.07 | 0.89 |

**Supplementary Table 3 |** Sediment concentrations of 16 HAP components at the 8 studied sites.

| **Site** | **Naphthalene** | **Acenaphthylene** | **Acenaphthene** | **Fluorene** | **Phenanthrene** | **Anthracene** | **Fluoranthene** | **Pyrene** | **Benzo (a) anthracene** | **Chrysene** | **Benzo (b) fluoranthene** | **Benzo (k) fluoranthene** | **Benzo (a) pyrene** | **Indeno (1,2,3-cd) pyrene** | **Dibenzo (a,h) anthracene** | **Benzo (g,h,i) perylene** |
| --- | --- | --- | --- | --- | --- | --- | --- | --- | --- | --- | --- | --- | --- | --- | --- | --- |
|  | (µg kg^-1^ dw) | | | | | | | | | | | | | | | |
| 5 | 7.7 | 2.5 | 2.9 | 4.8 | 62.9 | 14.3 | 157.9 | 142.0 | 82.4 | 114.8 | 163.0 | 63.4 | 110.4 | 78.3 | 19.5 | 94.2 |
| 6 | 12.9 | 3.6 | 4.4 | 1.3 | 79.4 | 15.2 | 196.9 | 164.0 | 77.2 | 132.1 | 205.4 | 80.8 | 126.0 | 120.4 | 28.5 | 124.2 |
| 21 | 9.6 | 2.9 | 3.7 | 0.6 | 69.7 | 13.0 | 162.3 | 132.7 | 60.2 | 114.6 | 174.8 | 61.7 | 106.1 | 101.7 | 23.0 | 102.8 |
| 29 | 9.1 | 2.9 | 6.3 | 9.5 | 98.0 | 22.0 | 281.0 | 235.6 | 124.6 | 201.3 | 276.1 | 110.9 | 197.6 | 150.5 | 37.5 | 159.2 |
| 32 | 18.5 | 2.7 | 5.6 | 39.7 | 107.4 | 24.5 | 325.6 | 296.2 | 156.4 | 234.7 | 340.6 | 137.1 | 212.8 | 163.7 | 41.8 | 155.0 |
| 36 | 112.4 | 32.7 | 59.0 | 104.8 | 853.4 | 861.3 | 1908.8 | 1584.2 | 1253.9 | 1691.1 | 1456.9 | 765.7 | 1391.8 | 657.0 | 241.8 | 606.4 |
| 53 | 425.6 | 22.3 | 238.2 | 2848.1 | 2159.0 | 467.3 | 3441.4 | 3795.4 | 1851.8 | 2585.3 | 2522.5 | 893.5 | 1720.9 | 1079.6 | 274.4 | 936.8 |
| 78 | 19.0 | 23.3 | 2.0 | 146.5 | 70.8 | 17.3 | 93.8 | 131.2 | 39.4 | 58.5 | 55.5 | 18.1 | 43.3 | 23.4 | 4.4 | 27.8 |

**Supplementary Table 4 |** Environmental variables showing significant correlations with the procrustean analysis community ordination.

| **Environmental variable** | **Label** | **r^2^** | **P** |
| --- | --- | --- | --- |
| Carbonate content | Carb | 0.815 | < 0.001 |
| Total organic carbon | TOC | 0.902 | < 0.001 |
| Total phosphorus | Ptot | 0.990 | < 0.001 |
| Median grain size | Grain Size | 0.831 | < 0.001 |
| Titanium concentration | Ti | 0.942 | < 0.001 |
| Chrome concentration | Cr | 0.771 | < 0.001 |
| Arsenic concentration | As | 0.861 | < 0.001 |
| Sum of the other trace metal concentrations | Σ other trace metals | 0.976 | < 0.001 |
| Sum of the 7 indicator PCB concentrations | Σ PCBi | 0.990 | < 0.001 |
| Sum of the PAH concentrations | Σ PAH | 0.854 | < 0.001 |
